# Global Advancements in Bioactive Material Manufacturing for Drug Delivery: A Comprehensive Study

**DOI:** 10.1021/acsomega.4c08669

**Published:** 2025-01-03

**Authors:** Rafael Leandro Fernandes Melo, Dayana Nascimento Dari, Francisco Izaias da Silva Aires, Francisco Simão Neto, Tiago Melo Freire, Bruno Caio Chaves Fernandes, Pierre Basílio Almeida Fechine, João Maria Soares, José Cleiton Sousa dos Santos

**Affiliations:** † Departamento de Engenharia Metalúrgica e de Materiais, Universidade Federal do Ceará, Campus do Pici, Bloco 729, Fortaleza CEP 60440-554, CE, Brazil; ‡ Grupo de Química de Materiais Avançados (GQMat), Departamento de Química Analítica e Físico-Química, Universidade Federal do Ceará, Campus do Pici, Fortaleza CEP 60451-970, CE, Brazil; § Grupo de Engenharia e Desenvolvimento Sustentável (GENES), Instituto de Engenharias e Desenvolvimento Sustentável, Universidade da Integração Internacional da Lusofonia Afro-Brasileira, Campus das Auroras, Redenção CEP 62790-970, CE, Brazil; ∥ Departamento de Engenharia Química, Universidade Federal do Ceará, Campus do Pici, Bloco 709, Fortaleza 60455-760, CE, Brazil; ⊥ Departamento de Agronomia e Ciência Vegetais, Universidade Federal Rural do Semi-Árido, Campus Mossoró, Mossoró CEP 59625-900, RN, Brazil; # Departamento de Física, Universidade do Estado do Rio Grande do Norte, Campus Mossoró, Mossoró CEP 59610-090, RN, Brazil

## Abstract

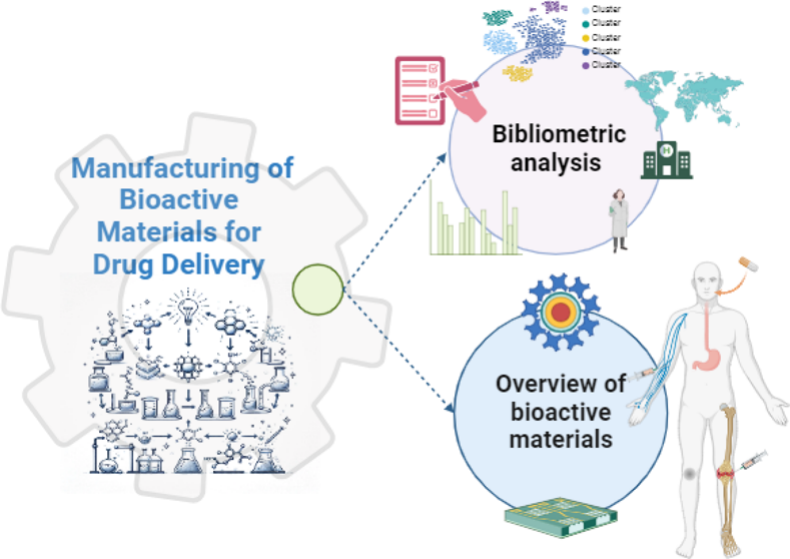

Manufacturing bioactive materials for drug delivery involves developing materials that interact with biological tissues to release drugs in a controlled and targeted manner. The goal is to optimize therapeutic efficacy and reduce side effects by combining knowledge from materials engineering, biology, and pharmacology. This study presents a detailed bibliometric analysis, exploring the keywords “manufacturing,” “bioactive materials,” and “drug delivery” to identify and highlight significant advancements in the field. From the Web of Science, 36,504 articles were analyzed, with 171 selected for a deeper analysis, identifying key journals, countries, institutions, and authors. The results highlight the field’s interdisciplinary nature, with keywords grouped into four main themes, including regenerative medicine, scaffolds, three-dimensional (3D) printing, bioactive glass, and tissue engineering. Future research in this area will focus on more effective and precise systems using technologies like 3D printing and nanotechnology to enhance the customization and control of drug release, aiming for more efficient and targeted therapies.

## 1 Introduction

The manufacture of bioactive materials represents an innovative and multidisciplinary field at the forefront of biomedical research and materials engineering.^1−3^ This domain focuses on developing substrates and systems capable of interacting positively with biological systems to facilitate or enhance drug delivery.^4−6^ The importance of this research area has grown exponentially as demands for more effective and less invasive therapies continue to rise in response to the ongoing challenges of modern medicine.^7,8^

The central goal of manufacturing bioactive materials for developing drug delivery systems is to create platforms that can be precisely controlled and directed to target specific locations within the body, releasing the therapeutic agent in a controlled and sustained manner.^9−11^ These platforms range from nanoparticles to hydrogels and three-dimensional (3D) scaffolds, each offering unique mechanisms to interact with target cells and tissues and respond to specific stimuli.^12−14^

The integration of advanced manufacturing technologies, such as 3D printing and nanotechnology, has enabled unprecedented advances in the precision and functionality of bioactive materials.^15,16^ These technologies facilitate the production of complex structures at nanometric scales, allowing the incorporation of functional features that enhance the efficacy of drug delivery and biocompatibility.^17−19^

This paper highlights the importance of conducting a comprehensive bibliometric review focused on manufacturing bioactive materials to develop drug delivery systems. Through analyzing publication patterns, collaborations between authors and institutions, the geographical distribution of research, and the evolution of topics of interest over time, it is possible to gain valuable insights into technological advances, existing knowledge gaps, and potential areas for innovation.^20−22^

Manufacturing bioactive materials for drug delivery is characterized by its interdisciplinary nature, bringing together knowledge from biology, chemistry, physics, materials engineering, and pharmaceutical sciences.^23−26^ Given the vast scope of this field, a bibliometric review offers a systematic and quantitative perspective that can help researchers, policymakers, and investors better understand current dynamics and make informed decisions about allocating resources and research efforts.^27−29^

By mapping the current landscape and anticipating future trends in manufacturing bioactive materials for drug delivery, this article provides a solid foundation for future investigations and developments.^30−32^ It is expected that this bibliometric analysis will contribute to accelerating advances toward more innovative and effective therapeutic solutions by illuminating the paths taken and the promising horizons.

## 2 Methodology

**Figure 1:**
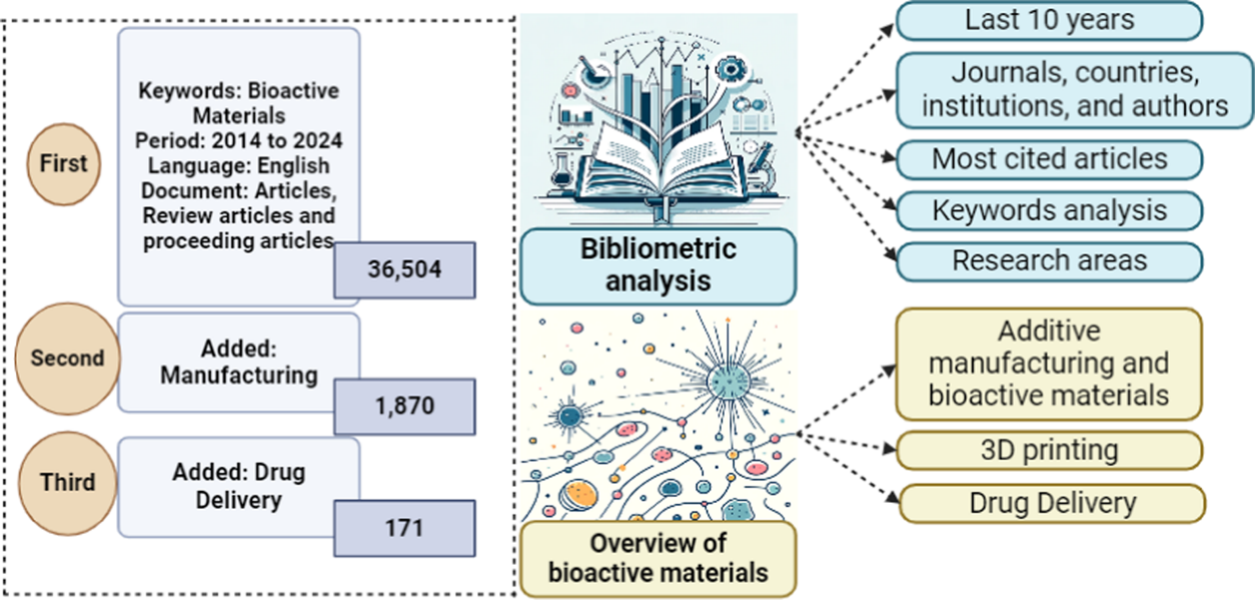
Methodological steps for the bibliometric analysis.

A bibliometric investigation was conducted, supported by prior literature.^33−36^ The methodology adopted for this analysis is detailed in Figure 1, describing the bibliographic and bibliometric study process. We used the Web of Science (WoS) database to collect a comprehensive set of publications on the topic, filtering for articles and reviews published between 2014 and 2024. The specific keywords for the search were “Bioactive Materials,” “Manufacturing,” and “Drug Delivery,” resulting in the selection of 171 articles for bibliometric analysis.

The selected keywords were obtained through exhaustive testing of the best keyword combinations that yielded the highest results in WoS. Other attempts produced fewer results and failed to form adequate collaboration networks. Some limitations are inherent to bibliometric review articles, such as the influence of coauthors, which can affect the analyzed connections, self-citations, which may inflate impact indicators, and the inclusion of other review articles, which can distort results by synthesizing multiple studies. However, when these issues are addressed rigorously, bibliometric reviews provide a reliable and comprehensive view of trends and impact in the researched area, as demonstrated by numerous studies published using this methodology.^37−40^

To decipher the trends and patterns within this vast corpus, we posed questions such as the evolution of research on the manufacture of bioactive materials for achieving drug delivery over the last 10 years; the prominent journals, countries, institutions, and authors involved; the most referenced works; the frequently used keywords; and the predominant fields of publication.

The analysis of the articles was facilitated by the use of two main bibliometric tools: VOSviewer (version 1.6.18), accessible at https://www.vosviewer.com/, to create network maps illustrating the connections between journals, countries, institutions, authors, and the most prominent keywords in the field; and the Bibliometrix extension (version 3.0) for R Studio, available at http://www.facebook.com/en/libr. https://www.bibliometrix.org/, applied to generate the three-field plot and bibliometric ranking tables, and analyze the emerging thematic clusters.

The conclusion of this study presents an overview of the manufacture of bioactive materials for drug delivery.

## 3 Results and Discussion

### 3.1 Bibliometric Analysis of the Manufacture of Bioactive Materials for Drug Delivery in the Last 10 Years

**Figure 2:**
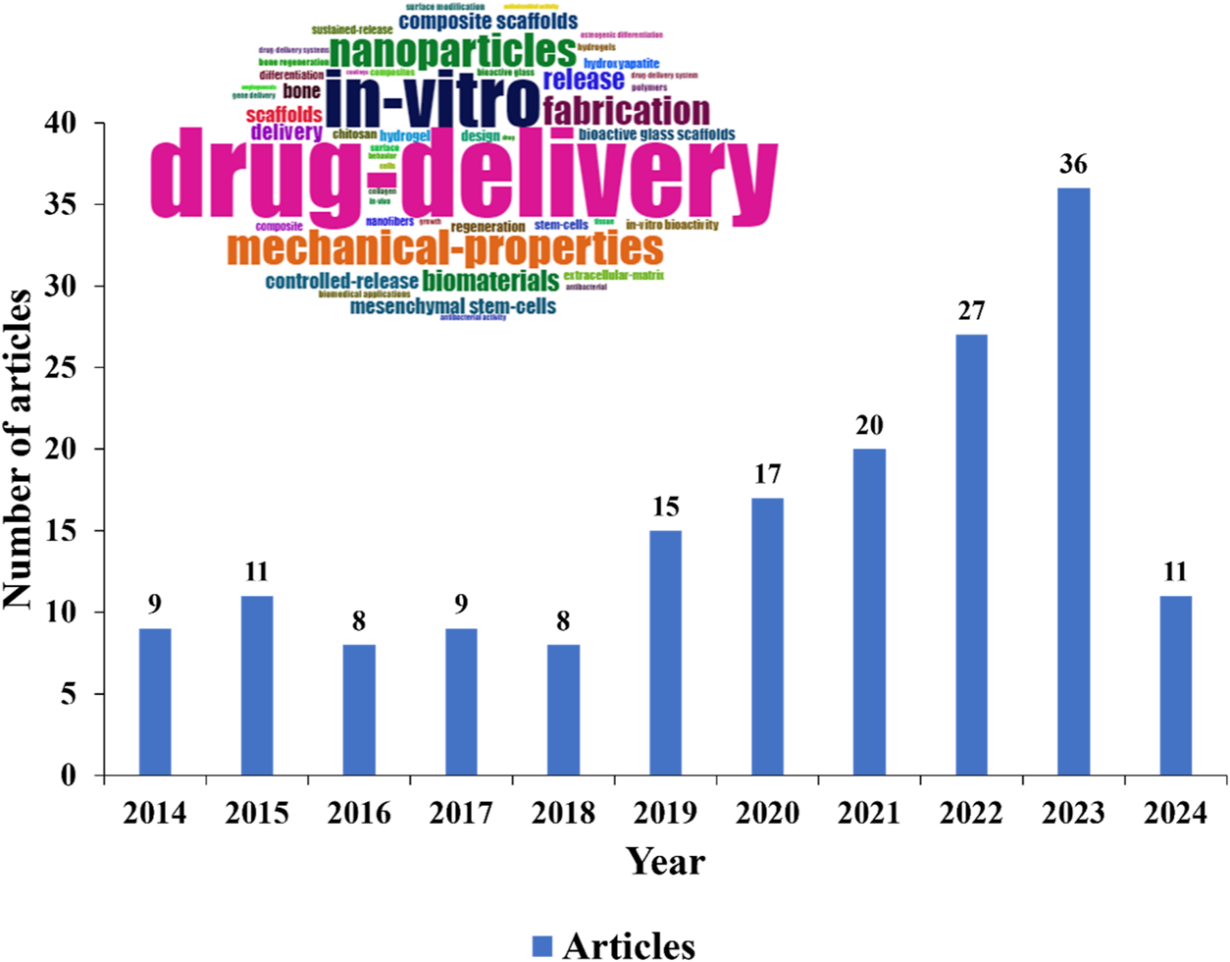
Evolution of the number of publications from 2014 to 2024.

Figure 2 illustrates the trend in publications on manufacturing bioactive materials for drug delivery from 2014 to 2024. There has been an overall increase in publications over the years, despite a brief reduction between 2016 and 2018. The peak occurred in 2023, with 36 publications indicating a growing interest in this area. However, it is still considered a niche field compared to broader topics such as bioactive materials, which recorded 932 publications in 2024. In 2014, there were nine publications in this field, with the most cited (62 citations) being a review article in the “Journal of Biomedical Materials Research.” This article highlighted the advancement and application of nano/microfibrous polymeric constructions in tissue engineering, using electrospinning and additive manufacturing (AM) techniques to replicate the extracellular matrix.^41^ In 2023, the article with the highest number of citations (41 citations), published in “Bioactive Materials,” focused on the progress and applications of nano/microfibers in the biomedical field. It includes topics such as tissue engineering, drug delivery, wound healing, and biosensors, highlighting these fibers’ innovative manufacturing technologies and unique properties.^42^ In 2024, a decrease in the number of articles is observed, possibly because the year has not yet concluded. The word cloud in the graph highlights the most frequent keywords in the articles over the last 10 years, including terms such as “drug delivery,” “in vitro,” “mechanical properties,” “nanoparticles,” and “fabrication.”

#### 3.1.1 Study of Journals, Countries, Institutions, and Authors

Table 1 ranks the top 10 journals, countries, institutions, and authors in terms of the number of publications on the manufacture of biomaterials for drug delivery over the last 10 years. “Journal of Materials Chemistry B″ leads in the number of publications, accumulating 254 citations with an AC index (citations per publication) of 28.22. The most cited study from this journal, with 53 citations, discusses the development of bioactive glass nanoparticles (BGNs) doped with europium (BGN-Eu) for use in drug delivery and cellular imaging, highlighting their biocompatibility and potential in bone regeneration and cellular marking with fluorescence.^43^ “Acta Biomaterialia” leads with an AC index of 65.33 and only three publications. The article for this index is titled “Surface modification of 3D-printed porous scaffolds via mussel-inspired polydopamine and effective immobilization of rhBMP-2 to promote osteogenic differentiation for bone tissue engineering,” which received 146 citations. The article focuses on developing 3D-printed polycaprolactone structures for bone regeneration, grafted with rhBMP-2 via polydopamine chemistry, showing improved hydrophilicity, cell attachment, and osteoconductivity.^44^

**Table 1:** Ranking the 10 Prominent Journals, Countries, Institutions, and Authors from the Last 10 Years with the Highest Number of Publications in Manufacturing Bioactive Materials for Drug Delivery^a^

ranking		NP	NC	AC
Journal				
1	Journal of Materials Chemistry B	9	254	28.22
2	Materials	9	466	51.78
3	Bioactive Materials	7	320	45.71
4	Biomedical Materials	4	216	54.00
5	Journal of Drug Delivery Science and Technology	4	57	14.25
6	Materials Today Bio	4	27	6.75
7	Pharmaceutics	4	14	3.50
8	ACS Applied Materials & Interfaces	3	93	31.00
9	Acta Biomaterialia	3	196	65.33
10	Advanced Healthcare Materials	3	103	34.33
Country				
1	China	48	1014	21.13
2	USA	32	1586	49.56
3	England	21	718	34.19
4	India	17	476	28.00
5	Iran	17	734	43.18
6	Italy	16	472	29.50
7	Spain	13	501	38.54
8	South Korea	9	287	31.89
9	Germany	8	421	52.63
10	Australia	7	286	40.86
Institutions				
1	Universidade Jiaotong de Xiam	9	247	27.44
2	Zhejiang Sci-Tech University	7	142	20.29
3	Chinese Academy of Sciences	6	143	23.83
4	Tabriz University of Medical Sciences	5	381	76.20
5	UCL—London’s Global University	5	118	23.60
6	Universidad Complutense de Madrid	5	354	70.80
7	National University of Singapore: NUS	4	133	33.25
8	Harvard Medical School	3	79	26.33
9	Isfahan University of Medical Sciences	3	121	40.33
10	Queen Mary University of London	3	66	22.00
Authors				
1	Lei, Bo	8	246	30.75
2	Jiang, Guohua	4	75	18.75
3	Vallet-Regi, Maria	4	353	88.25
4	Wang, Min	4	122	30.5
5	Ma, Peter X.	3	162	54
6	Razavi, Mehdi	3	63	21
7	Tappa, Karthik	3	91	30.33
8	Wang, Tao	3	44	14.67
9	Wang, Xiaohong	3	70	23.33
10	Xue, Yumeng	3	162	54

a NP = number of publications; NC = number of citations; AC = average citations (NC/NP).

China led in the number of publications, followed by the United States and England, with 48, 32, and 21 publications, respectively. However, regarding the AC (citation per publication) factor, China ranked behind the other nine countries with only an AC of 21.13. Germany, which has only eight publications, led the AC index with 52.63.

The institution with the highest number of publications is Xi’an Jiaotong University, which has nine publications, followed by Zhejiang Sci-tech University, which has seven publications. Tabriz University of Medical Sciences recorded the highest AC factor, at 76.20.

Lei, Bo leads with the most publications, totaling nine, while Jiang, Guohua, Vallet-Regi, Maria, and Wang, Min each have four. Vallet-Regi, Maria stands out with the highest number of citations at 353 and an AC factor of 88.25. The most cited article by Lei, Bo, with 66 citations, was published in 2017 in “ACS Applied Materials & Interfaces.” It is similar to the most cited article in “The Journal of Materials Chemistry B.″ It discusses the properties of bioactive glass nanoparticles (BGNs) as safe and effective vectors for drug and gene delivery. The article highlights that BGNs have a high binding capacity for drugs and miRNA without the need for modifications with cationic polymers, showing advantages in biocompatibility, biodegradability, drug loading capacity, and transfection efficiency compared to other inorganic nanoparticles and commercial reagents.^45^

**Figure 3:**
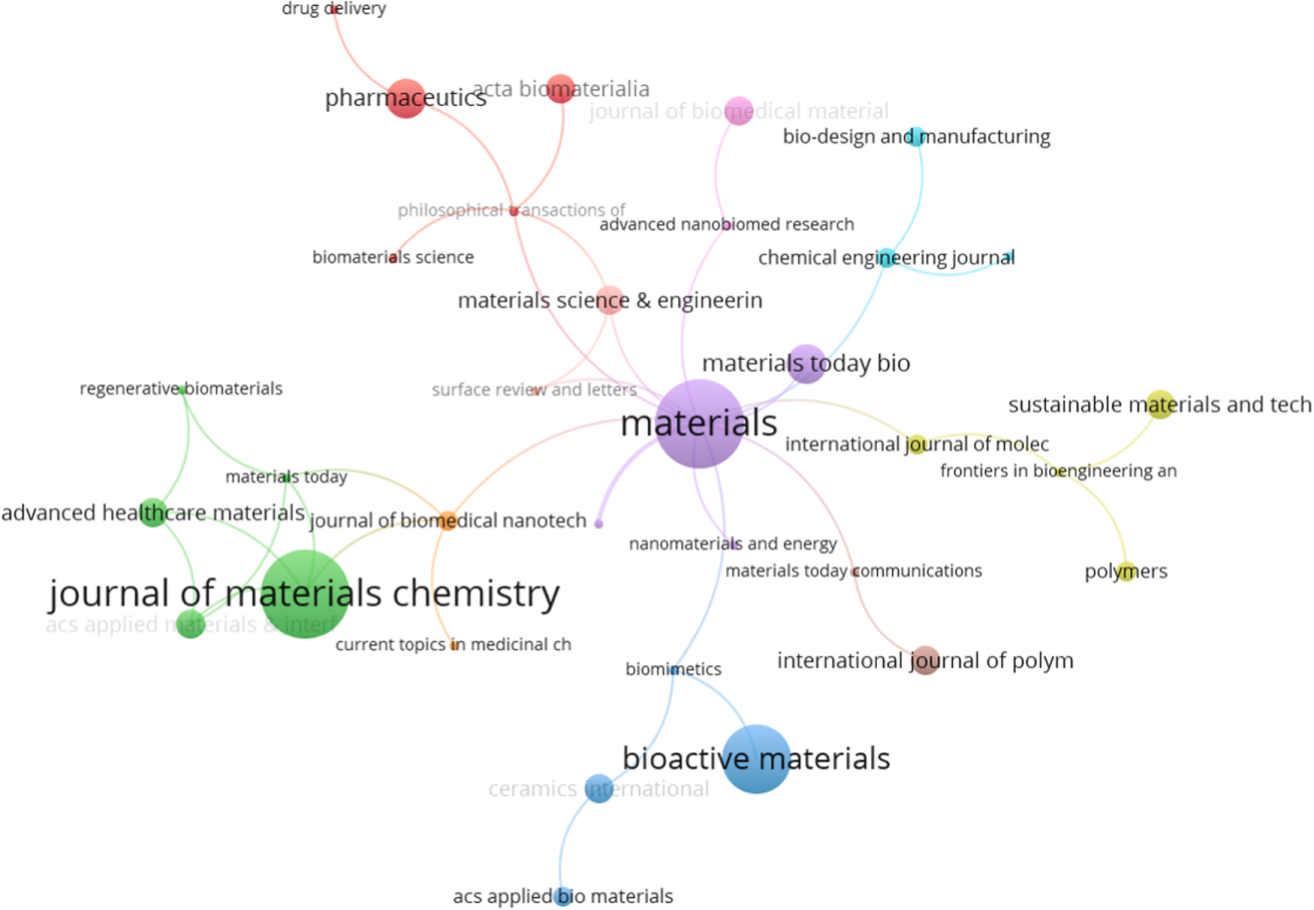
Bibliometric analysis of research on the manufacture of bioactive materials for drug delivery. Ranking of the top 10 journals with the most publications in the area.

Figure 3 shows the network analysis formed by VOSviewer of the most significant journals, with the related research terms “bioactive materials,” “manufacture,” and “drug delivery.”

We observe that the journals “Journal of Materials Chemistry,” “Materials,” and “Bioactive Materials” (green, purple, and blue clusters, respectively) are at the center of the network, suggesting they are the prominent journals publishing relevant works that intersect these three domains. This may indicate that these periodicals are central to disseminating interdisciplinary research that advances the understanding and application of bioactive materials in drug delivery, an essential area in biomedical engineering and pharmacology.

The interconnection between these areas highlights the shift toward innovative tissue engineering and regenerative medicine approaches.

However, there is a need for a deeper analysis of how these diverse areas are being integrated. For instance, it is essential to question whether current research is sufficiently focused on the aspect of biocompatibility and the efficacy of bioactive materials. This approach will be addressed later in the article.

**Figure 4:**
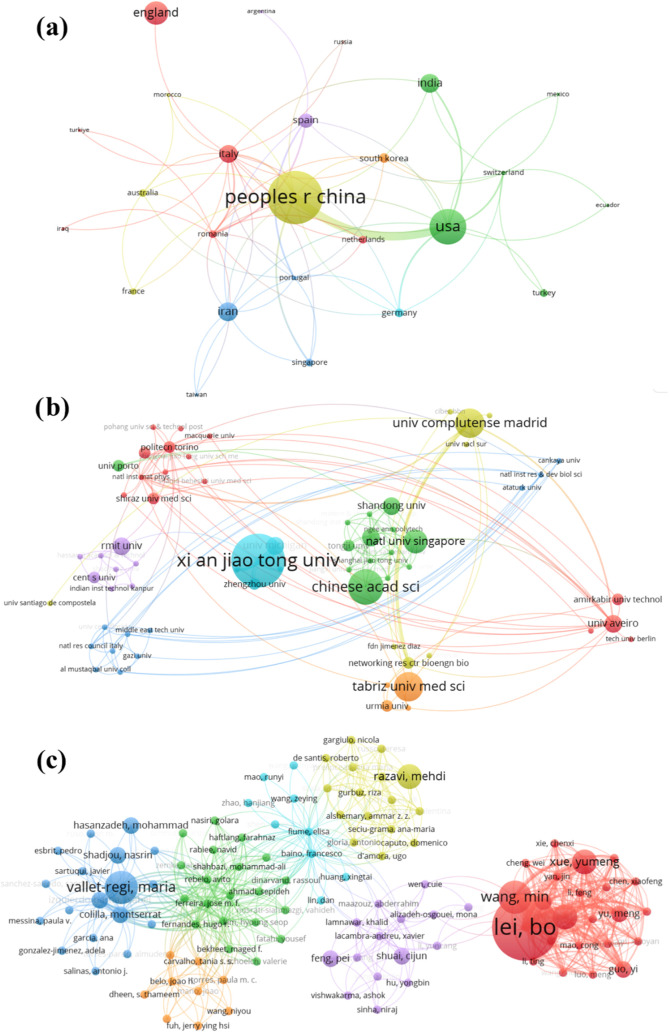
Bibliometric analysis of research on the manufacture of bioactive materials for drug delivery. (a) Network analysis of the most influential countries. (b) Network analysis of the most influential institutions. (c) Network analysis of the most influential authors.

The network analysis presented in Figure 4 is a powerful tool for visualizing the global research landscape involving bioactive materials, manufacturing, and drug delivery. Figure 4a displays the representation of countries, underscoring the importance of international collaboration and geographical distribution in advancing research, with China, the USA, and England emerging as significant leaders. This likely reflects their investment in research and the priority given to these areas. China is shown as the country with the most publications (indicated by the yellow cluster), followed by the USA (green cluster) and England (red cluster).

Figure 4b delves into the institutional sphere, mapping the leading institutions in publications. The dominance of universities such as Xi’an Jiaotong University (blue cluster) shows where research is concentrated, and potential partnerships for future collaborations can be developed. This emphasizes the importance of such centers as catalysts for innovation and education.

Figure 4c provides a focused view, focusing on the individual contributors to the research. The analysis of the most productive authors acknowledges their contribution to the field and may also indicate the knowledge networks and mentorship essential for the ongoing development of biomaterials research. Lei, Bo stands out in a well-defined cluster (red cluster), encompassing several other authors, indicating that his research is collaborative and interconnected.

These analyses are crucial for understanding the flow of collaboration and knowledge in the research of bioactive materials for drug delivery. They reveal patterns of publication and citation and suggest areas for productive investment. However, despite being quantitative, it is necessary to assess the quality and impact of the publications for a complete view of the progress in the field. This point will be further explored throughout this article.

**Figure 5:**
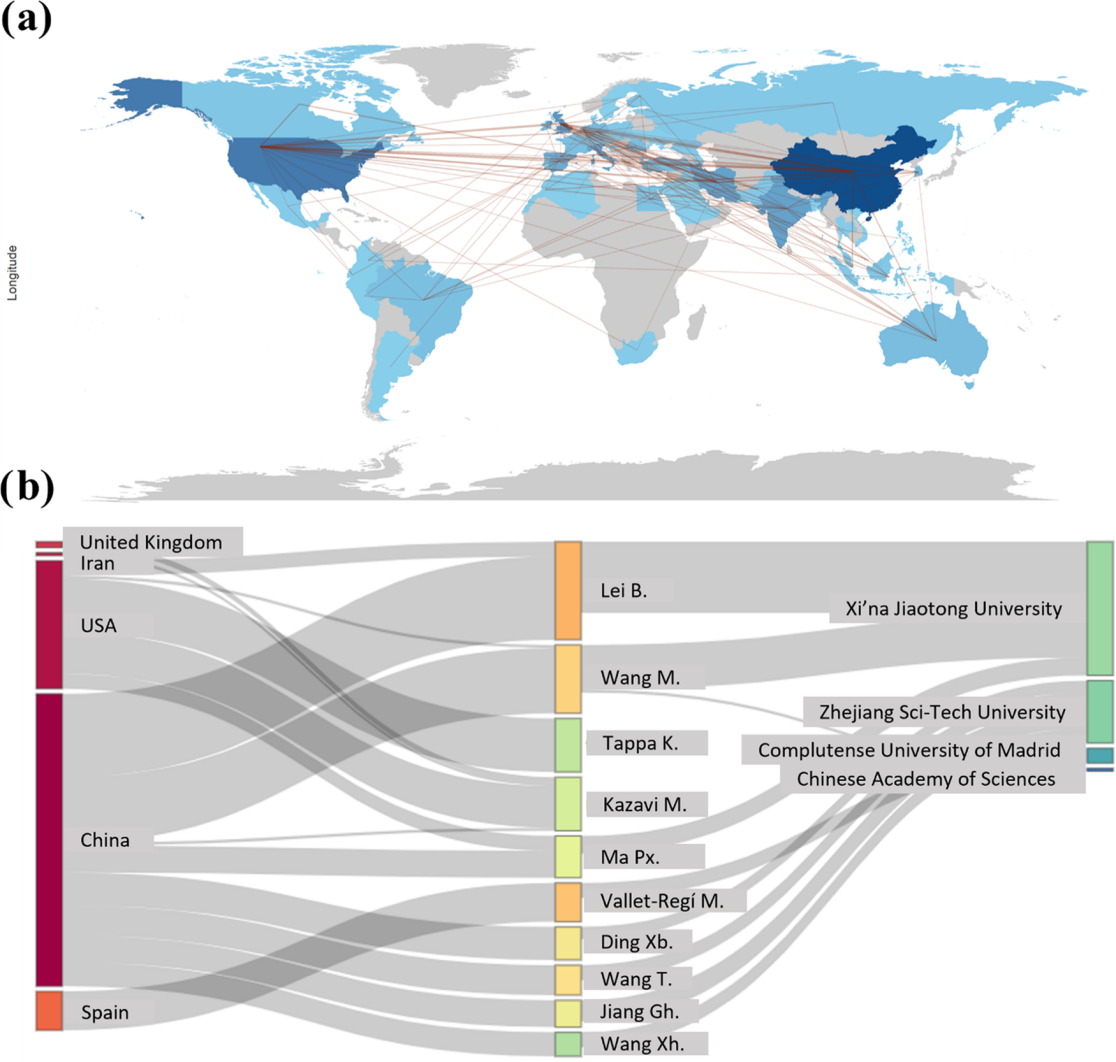
Bibliometric analysis of research on the manufacture of bioactive materials for drug delivery. (a) Cooperation map shows collaborations between all countries published in this area. (b) Sankey graph showing the interconnection between countries, authors, and institutions.

Figure 5 provides a comprehensive visual representation that captures the global and interconnected nature of bioactive materials, manufacturing, and drug delivery research. Figure 5a features a world map illustrating the connections between countries, highlighting the flow of international collaboration. Countries such as China, the USA, and the United Kingdom are significant centers of academic production, likely reflecting their substantial investment in research, as corroborated by Figure 4a.

Figure 5b features a Sankey diagram demonstrating the relationship among countries, authors, and institutions, emphasizing individual contributions to the overall body of research. For example, Lei, Bo from Xi’an Jiaotong University is notable for the volume of work produced and the impact within the scientific network, as demonstrated.

Therefore, Figure 5 is a statistical representation and a map of the collaborative framework that propels the field forward. Recognizing the interdependence of these connections is essential when analyzing coauthorship networks in biomedical research. This map can guide future collaboration strategies and help identify potential research gaps.

#### 3.1.2 Most Cited Articles in the Research Field

Table 2 presents the 10 most cited articles on advances in manufacturing bioactive materials for drug delivery over the past 10 years. The main themes related to this topic were identified through the analysis of these publications. The most cited article, published in 2018 by the journal “Progress in Materials Science, has accumulated 417 citations. It reviews the use of biomaterials in the functional restoration engineering of different tissues, presenting the leading additive manufacturing methods and their applications in various treatment modalities.^46^ Another relevant article from the same journal, ranked as the fourth most cited with 210 citations, is one of the most recent. It discusses using chitosan, a natural biopolymer, in biomedical applications, highlighting its advantageous properties, such as biocompatibility, biodegradability, and antibacterial activity. This article focuses explicitly on the electrophoretic deposition (EPD) technique of chitosan, especially in combination with other materials, to create composite coatings.^47^

**Table 2:** Most Cited Articles from the Last 10 Years

ranking	article title	authors	journal	year published	total citations	reference
1	additive manufacturing of biomaterials	Susmita Bose, Dongxu Ke, Himanshu Sahasrabudhe, Amit Bandyopadhyay	Progress in Materials Science	2018	417	(46)
2	microemulsions as carriers for drugs and nutraceuticals	Aviram Spernath, Abraham Aserin	Advances in Colloid and Interface Science	2006	268	(48)
3	3D printing of calcium phosphate ceramics for bone tissue engineering and drug delivery	Ryan Trombetta, Jason A. Inzana, Edward M. Schwarz, Stephen L. Kates, Hani A. Awad	Annals of Biomedical Engineering	2017	216	(49)
4	electrophoretic deposition of chitosan-based composite coatings for biomedical applications: a review	Egemen Avcu, Fatih E. Baştan, Hasan Z. Abdullah, Muhammad Atiq Ur Rehman, Yasemin Yıldıran Avcu, Aldo R. Boccaccini	Progress in Materials Science	2019	210	(47)
5	a comprehensive review of biodegradable synthetic polymer–ceramic composites and their manufacture for biomedical applications	Mona Alizadeh-Osgouei, Yuncang Li, Cuie Wen	Bioactive Materials	2019	185	(50)
6	bioactive glasses and glass-ceramics for healthcare applications in bone regeneration and tissue engineering	Hugo R. Fernandes, Anuraag Gaddam, Avito Rebelo, Daniela Brazete, George E. Stan, and José M. F. Ferreira	Materials	2018	171	(51)
7	poly(ε-caprolactone)/graphene oxide biocomposites: mechanical properties and bioactivity	Chaoying Wan, Biqiong Chen	Biomedical Materials	2018	169	(52)
8	calcium orthophosphate cements and concretes	Sergey V. Dorozhkin	Materials	2009	160	(53)
9	surface modification of 3D-printed porous scaffolds via mussel-inspired polydopamine and effective immobilization of rhBMP-2 to promote osteogenic differentiation for bone tissue engineering	Sang Jin Lee, Donghyun Lee, Taek Rim Yoon, Hyung Keun Kim, Ha Hyeon Jo, Ji Sun Park, Jun Hee Lee, Wan Doo Kim, Il Keun Kwon, Su A Park	Acta Biomaterialia	2016	146	(54)
10	recent advances on liposomal nanoparticles: synthesis, characterization and biomedical applications	Yunes Panahi, Masoud Farshbaf, Majid Mohammadhosseini, Mozhdeh Mirahadi, Rovshan Khalilov, Siamak Saghfi, Abolfazl Akbarzadeh	Artificial Cells, Nanomedicine, and Biotechnolog	2017	144	(55)

The second most cited article, published in the journal “Advances in Colloid and Interface Science,” accumulating 268 citations, explores microemulsions, which are dispersed systems of oil droplets in water or water in oil, stabilized by surfactants. Its potential as a vehicle for bioactive molecules is highlighted, mentioning advantages such as spontaneous formation, ease of manufacturing, and thermodynamic stability. Additionally, it discusses the solubilization of drugs, peptides, and nutraceuticals in microemulsions and their impact on permeability and bioavailability.^48^

The article ranked third, published in the journal “Annals of Biomedical Engineering” and with 216 citations, is a systematic review of manufacturing calcium phosphate (CaP) ceramics using the 3D printing technique. This article evaluates their in vitro biocompatibility, in vivo bone regenerative potential, and their use locally delivering bioactive molecules or cells.^49^

The fifth most cited article, published in the journal “Bioactive Materials” and accumulating 185 citations, is a review that addresses the growing application of materials in biomedical procedures with a particular focus on treating bone diseases and disorders using biodegradable polymer–ceramic composites. Various biomaterials, such as metals, ceramics, and polymers, are explored, and the challenges associated with using metals in implantable devices, such as toxicity and the stress shielding effect, are discussed. Furthermore, the use of hydroxyapatite coatings as a means to mitigate these issues is addressed.^50^

The sixth most cited article, with 171 citations, was published in the journal “Materials.” It explores the discovery of bioactive glasses (BGs) by Hench and his team in the late 1960s, driven by the need for implantable materials that could bond to living tissues. The Bioglas 45S5 is a prominent example of BG, capable of forming a hydroxyapatite layer similar to bone when in contact with biological fluids. However, the article also points out the limitations of this material, such as potential cytotoxicity due to high sodium content and difficulty in manufacturing porous three-dimensional structures.^51^

The seventh-ranked article, published in the journal “Biomedical Materials” and accumulating 169 citations, explores the biomedical applications of graphene, a material that has received considerable attention recently. The focus is on graphene-based nanomaterials and their potential application in drug delivery, biosensors, and bioimaging areas. The cited study investigates the mechanical properties and bioactivity of nanofibrous and porous membranes made of graphene oxide (GO) nanoplatelets reinforced with poly(ε-caprolactone) (PCL). The results indicate that the addition of a small amount of GO to PCL membranes significantly increases their mechanical strength and enhances their bioactivity capacity during biomineralization processes.^52^

The eighth most cited article, with 160 citations, was also published in the journal “Materials” just like the sixth most cited. It addresses the discovery and development of calcium orthophosphate cement in powder and liquid forms, a bioactive and biodegradable graft material. These cements can harden and solidify within the body after being implanted, forming nonstoichiometric calcium-deficient hydroxyapatite (CDHA) or brushite, which are biocompatible and bioresorbable.^53^

The ninth most cited article, published in the journal “Acta Biomaterialia” and boasting 146 citations, addresses the manufacture and characterization of a 3D-printed porous scaffold made of polycaprolactone (PCL), a biocompatible material. The goal is to use this scaffold in bone tissue regeneration, aiming to create a hydrophilic structure that allows for cell attachment and is capable of binding bioactive molecules, such as bone growth factor-2, to promote cell differentiation.^54^

The 10th most cited article, published by the journal “Artificial Cells, Nanomedicine, and Biotechnology” with 144 citations, discusses the use of liposomes as nanostructures for encapsulating and delivering bioactive agents in various areas, such as cosmetics, food ingredients, and pharmaceuticals. The review presents the physicochemical properties of liposomes and manufacturing methods and presents some of their applications in food nanotechnology, including nutrient transport, enzymes, and antimicrobials. Additionally, it highlights the use of liposomes as drug carriers and gene delivery agents in biomedicine.^55^

A predominance of literature reviews is noted when analyzing the most cited articles in this bibliometric study, suggesting a solid foundation of original articles contributing to these reviews. These articles cover various topics in diagnostic and biomedical areas, reflecting the diversity of research and advances in these fields.

Studies on the use of biomaterials in tissue engineering stand out, emphasizing additive manufacturing methods and their applications in various treatments. Additionally, topics such as using chitosan in biomedical applications, the potential of microemulsions as vehicles for bioactive molecules, manufacturing calcium phosphate ceramics through 3D printing, and applying graphene in medicine are explored.

These articles reflect the growing interest and importance of these topics in the scientific community, significantly contributing to advancing knowledge and developing new technologies and therapies.

#### 3.1.3 Research Areas

**Figure 6:**
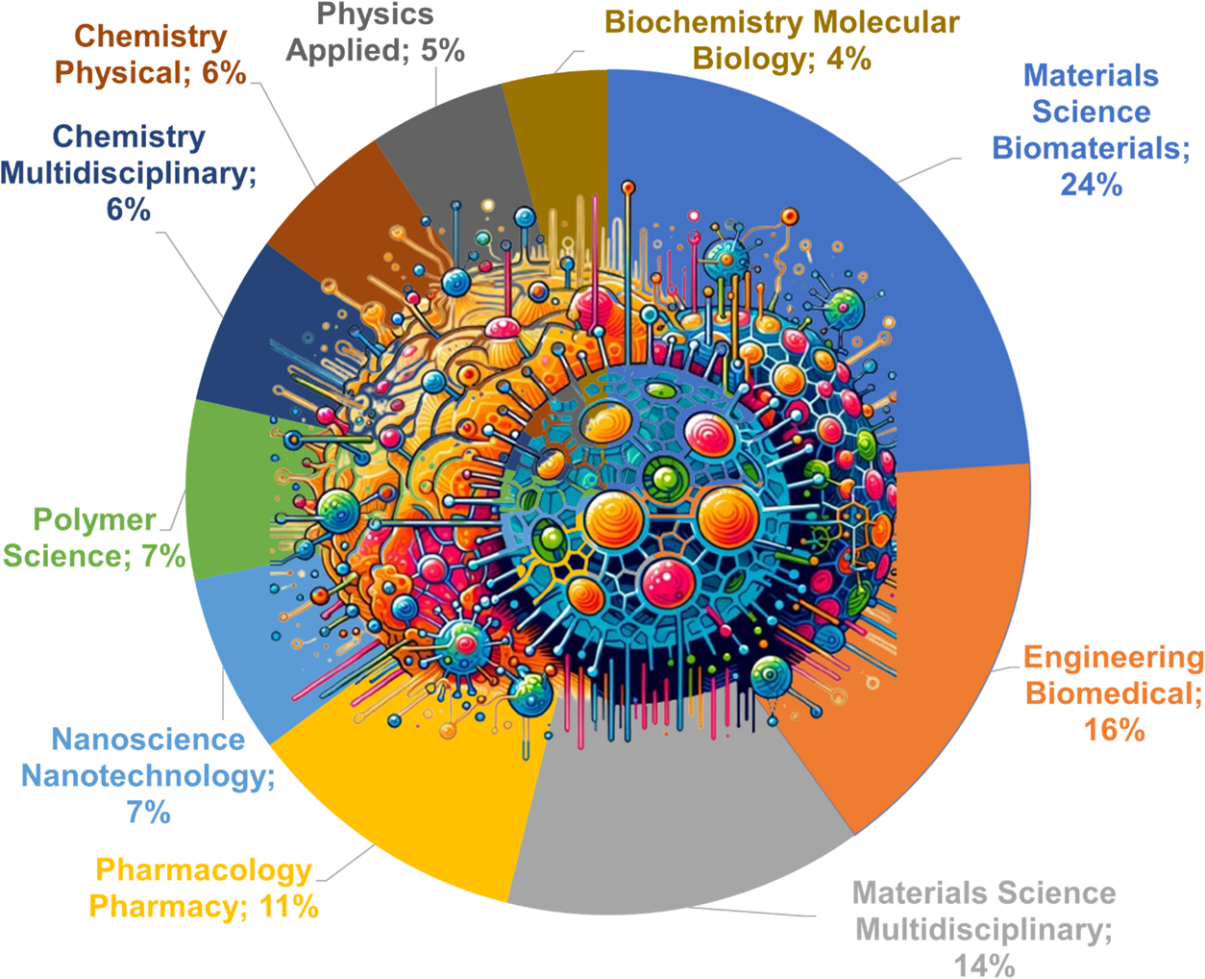
Distributions of research areas for articles on the manufacturing of bioactive materials for drug delivery.

Figure 6 illustrates the distribution of the top 10 research areas in advances in manufacturing bioactive materials for drug delivery over the past 10 years. The percentages have been adjusted to highlight the main themes based on data extracted from WoS. The Biomedical Materials area corresponds to 24%, followed by Biomedical Engineering at 16% and Multidisciplinary Materials at 14%.

The Biomedical Materials section encompasses a total of 59 articles. The most cited original article (146 citations) in this area aims to create porous scaffolds that are hydrophilic to allow for cell attachment and capable of binding a bioactive molecule to enhance cell differentiation.^54^

Biomedical Engineering presents a total of 34 articles. The most cited original article (41 citations) aims to develop and evaluate the effectiveness of three-dimensional (3D) scaffolds made with biodegradable polymers incorporating antibiotics interspersed with inorganic fillers manufactured by additive manufacturing (AM) for bone regeneration and infection prevention.^56^

The Multidisciplinary Materials area contains 34 articles. The most cited original research article (35 citations) aims to develop and investigate the effects of bioactive Si–Ca–Sr glass-based nanohybrids (BSr@PPE) in the context of wound healing impaired by tumor and infection, evaluating the effectiveness of these nanohybrids in killing tumor cells, combating multidrug-resistant bacteria, promoting fibroblast migration, and facilitating wound healing in vitro and in vivo models.^57^

An overlap of publications between the Biomedical Materials and Biomedical Engineering areas was observed, indicating related themes. Critical applications include developing materials for cell attachment and differentiation, bone regeneration, infection prevention, and the promotion of wound healing impaired by tumor and infection.

#### 3.1.4 Keyword Analysis

**Figure 7:**
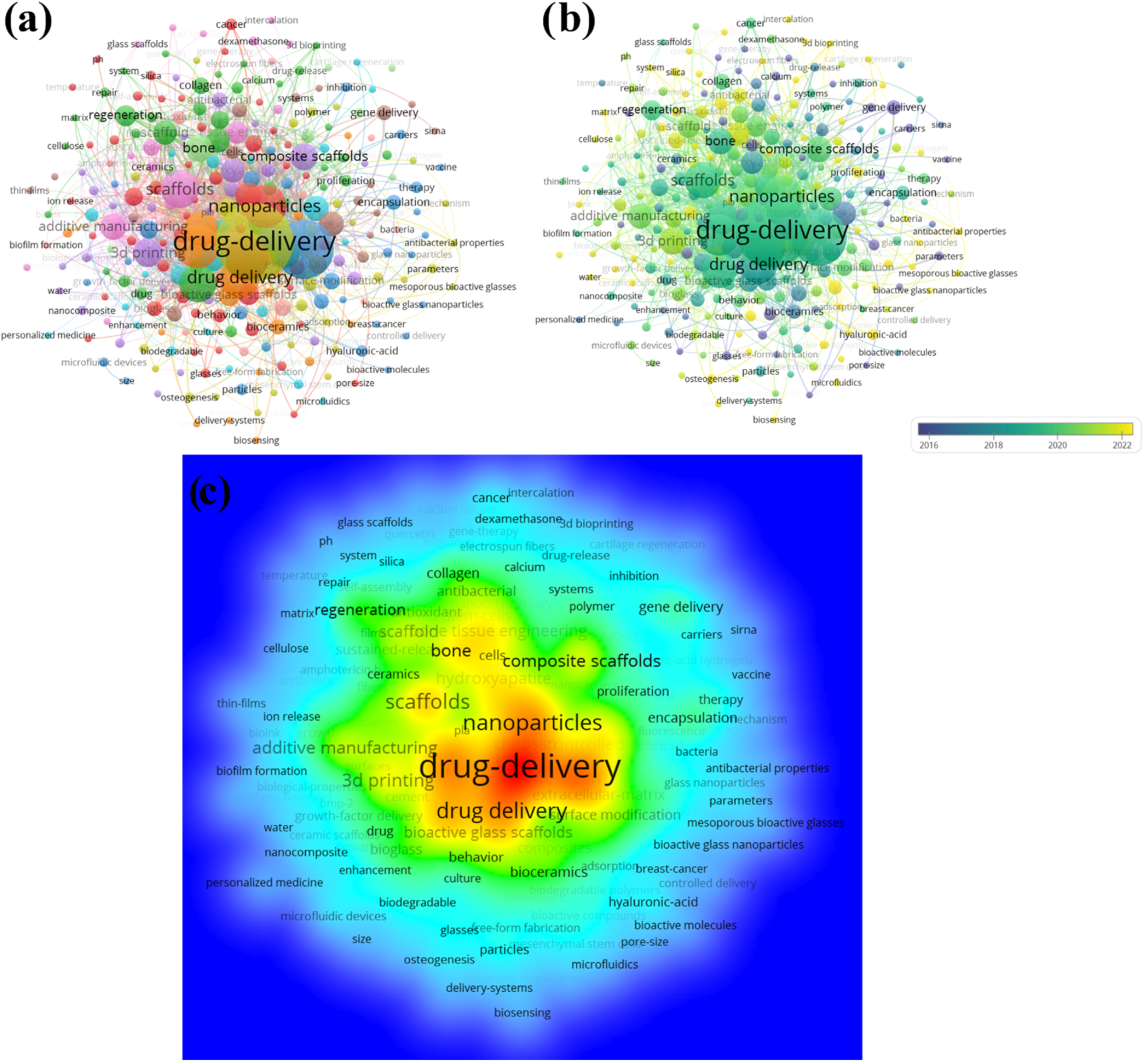
Network analysis of clusters formed by the main keywords related to manufacturing bioactive materials for drug delivery. (a) Clusters formed by the main keywords. (b) Temporal analysis of clusters. (c) Temperature gradient analysis of the clusters.

The bibliometric analysis also explored the most relevant keywords in the field, assisting in the identification of research clusters. Figure 7 presents the network analysis generated by VOSviewer, where six main clusters can be identified.

Figure 7a a collection in shades of pink stands out, centered on the keyword “scaffolds,” which has robust connections with other keywords such as “additive manufacturing,” “ceramics,” and “glass floors,” among others. The most cited articles in studies on bioactive materials for drug delivery and tissue engineering reveal a striking emphasis on using scaffolds. These three-dimensional structures play a crucial role in tissue regeneration and restoration.^58−60^

These scaffolds provide physical support for cells, facilitating their adhesion, proliferation, and differentiation in an environment similar to that of native tissue. Several studies address the development and application of scaffolds in various contexts, highlighting their importance in creating conducive environments for cell growth and facilitating regenerative therapies.^61,62^

In Figure 7a, a purple cluster is highlighted, with central terms such as “3D printing,” “composite scaffolds,” “antibacterial,” and “nanocomposite,” among others. The blue cluster groups keywords related to medicine and hospital treatments, while the green one presents terms such as “regeneration,” “cellulose,” “collagen,” and “drug release.” Finally, the brown, red, and yellow clusters represent sets of keywords related to drug release and biocompatibility.

In Figure 7b, the evolution of keywords over the years is observed. Initially, these were associated more with gene delivery and vaccines. Still, between 2018 and 2020, drug release and nanoparticles became widely recurring subjects, contributing to the significant advancement observed in these correlated areas.

In Figure 7c, the density of keywords in this bibliometric analysis can be visualized, excluding those used in this research. As can be seen, the keyword “drug release” exhibits the highest density.

Table 3 presents the ranking of the 20 most frequently found keywords in the articles, accompanied by their total link strength (TLS). In addition to the keywords mentioned in the previous topics, “in vitro,” “biomaterials,” and “mechanical Properties” stand out for their high TLS.

**Table 3:** Ranking of the 20 Most Prominent Keywords in the Analyzed Articles

rank	keyword	freq	TLS	rank	keyword	freq	TLS
1	drug delivery	74	158	11	3D printing	16	52
2	in vitro	48	125	12	bone	15	40
3	biomaterials	30	77	13	composite scaffolds	15	51
4	mechanical properties	30	82	14	additive manufacturing	14	48
5	nanoparticles	30	59	15	bone regeneration	14	39
6	drug delivery	27	72	16	controlled release	13	32
7	scaffolds	25	76	17	mesenchymal stem cells	13	28
8	fabrication	24	68	18	hydroxyapatite	12	36
9	tissue engineering	21	68	19	polymers	12	37
10	release	17	33	20	scaffold	12	41

**Figure 8:**
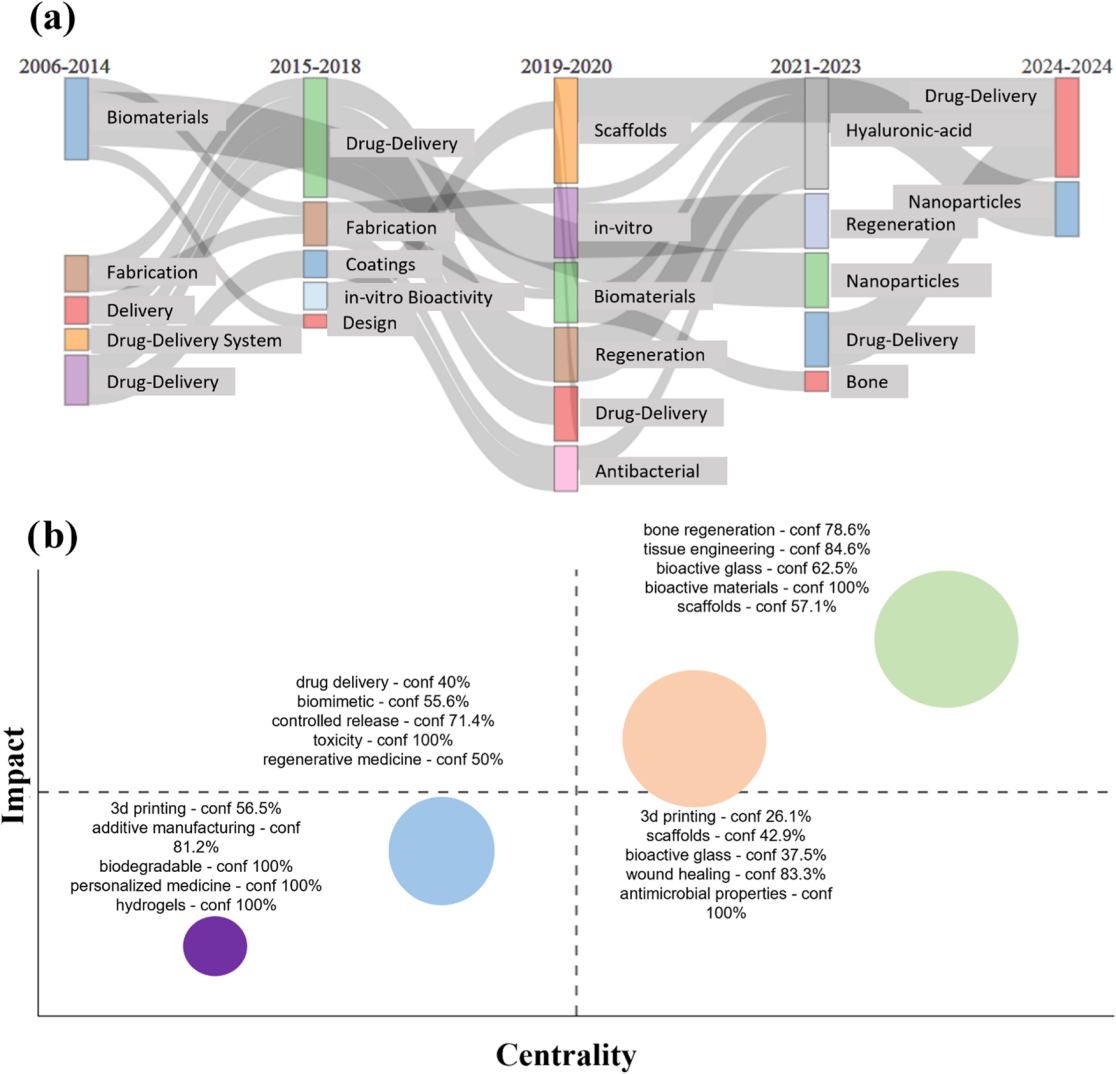
Bibliometric analysis of research on the manufacture of bioactive materials for drug delivery. (a) Evolution of the topic’s main keywords over the last 10 years. (b) Map of the main clusters correlating the impact and centrality of the topic.

Figure 8 was generated based on the data obtained by Bibliometrix. Figure 8a presents the variation in the frequency of keywords from 2014 to 2024 and their correlation in a five-field graph. By excluding the keywords used to construct the bibliometric analysis, we observe the emergence of terms such as “biomaterials,” “drug release,” and “drug delivery system” in the first field. Between the second and fifth groups, the keyword “drug release” stands out in all groups, and the emergence of the term “nanoparticles” between the fourth and fifth groups is noted. In the transition from 2023 to 2024, we noted a strong relationship between hyaluronic acid and the nanoparticles. Hyaluronic acid, a natural polysaccharide in the human body, is known for its biocompatibility and biodegradability, making it ideal for drug delivery systems.^63,64^

The recent integration of hyaluronic acid with nanoparticles offers a promising technique to enhance drug administration, particularly in targeted and regenerative therapies. Nanoparticles modified with hyaluronic acid allow for more precise delivery to cellular targets, increasing therapeutic efficacy by prolonging the circulation of the drug and reducing the immune response. This combination promotes the development of more effective and less invasive treatments, enabling advancements in personalized medicine and tissue regeneration.^65,66^

In Figure 8b, the formation of four clusters from the keywords is presented, highlighting their centrality and impact. All groups demonstrate different levels of centrality and effect. When organized in ascending order of centrality (excluding the keywords from this analysis), we identify the purple cluster, centered on “biodegradable,” “personalized medicine,” and “hydrogels,” followed by the blue cluster, which addresses “toxicity” and “controlled release.” Next, we have the orange cluster, encompassing “antimicrobial properties” and “wound healing.” Finally, the green cluster demonstrates the highest centrality associated with “bioactive materials” and “tissue engineering.” The exact sequence is observed when organized in ascending order of impact. The theme of bone regeneration appears to have a strong effect on the graph shown. Recent advancements in nanotechnology have ushered in innovative bone regeneration strategies, mainly through nanoparticles.^67,68^ These nanoparticles, engineered to specific sizes and surface properties, can deliver growth factors, proteins, or genetic materials directly to bone injury sites, thereby enhancing osteoinduction and osteoconduction.^69−71^ Nanoparticles coated with calcium phosphate or incorporated with bone morphogenetic proteins (BMPs) have shown significant potential in stimulating bone cell proliferation and differentiation.^72,73^ This targeted approach ensures the efficient delivery of therapeutic agents and minimizes systemic side effects, marking a significant leap forward in orthopedic regenerative medicine.^74^

Table 4 provides information on the identified clusters in Figure 8b, displaying the frequency of occurrence of each group and two representative articles that contributed to the formation of these clusters.

**Table 4:** Clusters Based on Bibliometric Analysis

cluster	main keywords	frequency	representations articles
1	drug delivery—conf 40% biomimetic—conf 55.6% controlled release—conf 71.4% toxicity—conf 100% regenerative medicine—conf 50%	9	(75−77)
2	bone regeneration—conf 78.6% tissue engineering—conf 84.6% bioactive glass—conf 62.5% bioactive materials—conf 100% scaffolds—conf 57.1%	31	(78−80)
3	3D printing—conf 26.1% scaffolds—conf 42.9% bioactive glass—conf 37.5% wound healing—conf 83.3% antimicrobial properties—conf 100%	28	(81) and (82)
4	3D printing—conf 56.5% additive manufacturing—conf 81.2% biodegradable—conf 100% personalized medicine—conf 100% hydrogels—conf 100%	6	(83−85)

**Figure 9:**
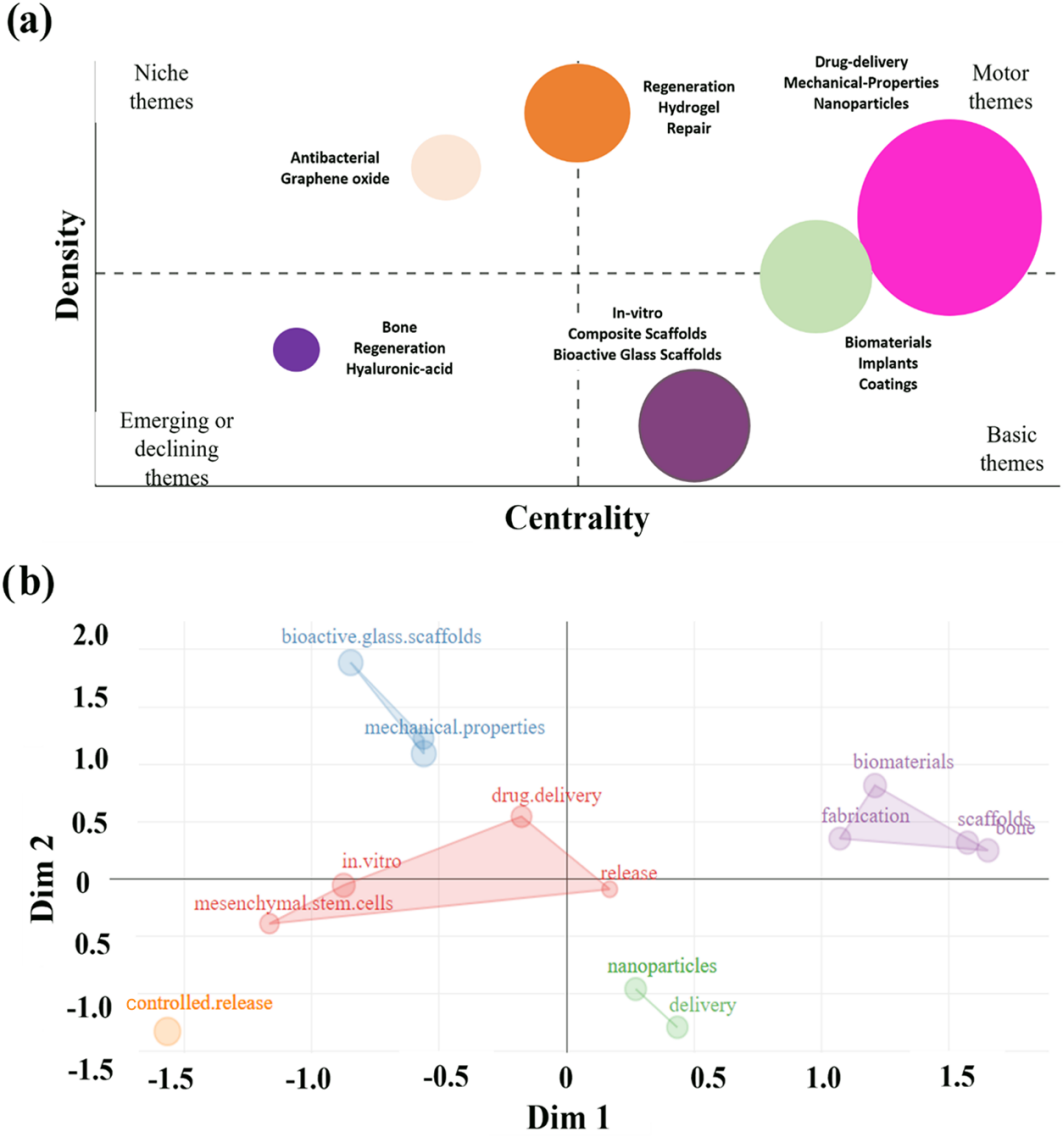
Bibliometric analysis of research on the manufacture of bioactive materials for drug delivery. (a) Thematic map correlating the themes into emerging, niche, driving, and basic themes. (b) Factor analysis of clusters.

Figure 9, derived from the data obtained by Bibliometrix, illustrates a cluster relationship in terms of centrality and keyword density, divided into four themes: emerging or declining, essential, niche, and driver.

In Figure 9a, the group with the highest centrality and the third highest density (in pink) is highlighted, positioned in the driver theme quadrant, where keywords such as “drug release,” “mechanical properties,” and “nanoparticles” are presented. There is also a cluster with the highest density and the fourth highest centrality (in orange), which includes keywords such as “regeneration.” Another group with the second highest centrality and the fourth highest density (in blue) contains words like “biomaterials.” The third highest centrality (in purple) group occupies the fundamental theme quadrant with the lowest density, highlighting keywords such as “in vitro.” The group with the fifth highest centrality and the second highest density (in pink) occupies the niche theme quadrant, highlighting keywords such as “antimicrobial properties.” The group with the fifth highest density (in pink) occupies the emerging or declining theme quadrant with the lowest centrality, highlighting keywords such as “regeneration.”

Figure 9b presents the map of the conceptual structure built through multiple correspondence analysis (MCA). The Porter stemming algorithm was used to reduce the number of words, considering only their basic forms or roots. The parameters employed in the factorial analysis included automatic clustering and a limit of 50 terms defined as the maximum number. The study revealed five distinct clusters, classified by size and represented in blue, green, red, purple, and orange.

The proximity of terms such as “drug.delivery,” “nanoparticles,” and “biomaterials” to each other indicates a significant overlap in research, reflecting the interdisciplinary trend in the application of nanoparticles and biomaterials in drug delivery systems. The term “mesenchymal.stem.cells” being close to “drug.delivery” suggests promising research using mesenchymal stem cells (MSCs) combined with drug delivery strategies.

Integrating mesenchymal stem cells (MSCs) with drug delivery strategies represents a frontier in regenerative medicine and targeted therapy. MSCs are renowned for their self-renewal capability and multipotency, with the ability to differentiate into various tissue types, offering a foundation for tissue repair.^86,87^ When combined with advanced drug delivery systems, such as nanoparticles, MSCs can be directed more effectively to the damaged tissue sites.^88,89^ This synergy between MSCs and precision drug delivery can enhance therapeutic outcomes by localizing treatment, reducing systemic side effects, and improving the regenerative process.^90,91^ The development of MSC-based drug delivery platforms is thus a promising avenue for improving patient outcomes in various degenerative diseases.

### 3.2 Overview of Bioactive Materials

#### 3.2.1 Additive Manufacturing and Bioactive Materials

Manufacturing additives and bioactive materials represents a crucial biomedical and pharmaceutical industry frontier.^92,93^ These materials are critical for precise drug delivery, especially in cancer research.^94^ Their ability to control drug release allows for a more targeted and practical approach to treating this disease. This may result in reduced side effects and improvements in the therapeutic efficacy. Additionally, these materials can be designed to target drugs to specific organs or target cells, increasing treatment efficiency and minimizing exposure of healthy tissues to therapeutic substances.^95,96^

However, there are challenges associated with manufacturing additives and bioactive materials.^97,98^ One of the main disadvantages is the complexity of formulating and manufacturing these materials, which often requires specialized and costly techniques. Furthermore, the safety and biocompatibility of these materials must be carefully evaluated to ensure that they do not cause adverse reactions in the body.^99,100^ Another challenge is ensuring the stability of materials during storage and transport, especially when dealing with sensitive medicines.^101,102^

Despite the challenges, advances in additive manufacturing and bioactive materials provide new opportunities for developing more effective personalized therapies.^103,104^ The ability to engineer materials that degrade in a controlled manner in the human body, gradually releasing drugs, is particularly promising for treating chronic diseases.^105,106^ Furthermore, nanotechnology and biotechnology in manufacturing these materials are paving the way for a new generation of drug delivery systems with greater precision and efficacy.^107,108^ In short, the manufacturing of additives and bioactive materials is a constantly evolving area of research that has the potential to significantly transform medical practice and improve patients’ quality of life.

#### 3.2.2 3D Printing

3D printing technology is emerging as a revolutionary tool in advancing the manufacturing of bioactive materials, driving innovations in engineering and medical applications.^109−111^ The advancement of three-dimensional technology offers opportunities to create innovative approaches and new methods in developing drug delivery systems.^112,113^ The ability to print complex structures with micrometer precision offers unique opportunities to design highly efficient, personalized drug delivery systems.^114^ The importance of this technology lies in its ability to produce specific shapes and personalized geometries that can improve therapeutic efficacy, minimize side effects, and facilitate drug administration.^115^

According to Qiu et al., 3D printing was initially used in the medical field to produce bioprostheses. However, over time, this technology has evolved significantly, expanding its applications to manufacture cells, tissues, organs, and medical robots, indicating a considerable diversification of its applications within medicine.^116^ 3D printing has become a highly versatile tool, standing out for its ability to produce complex structures with precision and adaptability.^117,118^

However, 3D printing in manufacturing bioactive materials for drug delivery also faces significant challenges.^119^ One of the dominant challenges is the large-scale economic production of microscale robotic devices. This barrier is currently increasingly important, especially when it comes to implants or implantable medical devices.^120^ Furthermore, obtaining materials with suitable mechanical and degradable properties can be complicated, requiring a deep understanding of materials chemistry.^121^ When contrasting pharmaceutical forms are produced by 3D printing with traditional ones, the gap in regulation and safety in areas that still lack complete solutions becomes evident, especially concerning the quality of materials and the techniques available for their monitoring.^122^

3D printing to manufacture bioactive materials includes the ability to produce personalized models of organs and tissues for preclinical testing, reducing the need for animal testing and accelerating the development of new medicines.^123−125^ Furthermore, the flexibility and versatility of 3D printing allow for the manufacturing of complex, personalized medical devices that can meet patient’s specific needs.^126,127^

In summary, 3D printing is a powerful tool in advancing bioactive materials for drug delivery, offering significant benefits in personalization, therapeutic efficacy, and cost reduction. However, challenges such as biocompatibility, printing accuracy, and material properties still need to be addressed to maximize the potential of this innovative technology in modern medicine.

#### 3.2.3 Drug Delivery

Drug delivery, especially about advances in manufacturing bioactive materials for drug delivery, is a critical field that seeks to optimize therapeutic efficacy and pharmaceutical index and minimize drug side effects.^128,129^ One of the main advantages of drug delivery is the ability to target medications to specific locations in the body, allowing for concentrated therapeutic doses where they are needed most. This may reduce systemic toxicity and improve treatment efficacy.^130,131^ Furthermore, controlled drug delivery can ensure sustained release over time, maintaining consistent therapeutic levels in the body and reducing the need for frequent administrations, minimizing possible side effects that may arise.^132^

However, drug delivery also presents significant challenges. One of the main challenges is the physiological barrier that can limit the absorption and distribution of drugs in the body, especially in specific locations such as the brain or tumors.^133−135^ Furthermore, selecting the appropriate delivery vehicle and drug formulation is essential to ensure drug stability during administration and transport to the site of action. Table 5 concisely summarizes the primary nanoparticles employed in drug delivery systems. It highlights the most common drugs these nanoparticles convey and assesses the advantages and disadvantages of each drug delivery method.

**Table 5:** Some of the Primary Nanoparticles and Drugs Used in Drug Delivery Systems

**nanoparticle**	**common drugs**	**advantages**	**disadvantages**	**references**
liposomes	doxorubicin, amphotericin B	biocompatible, carries both lipophilic and hydrophilic drugs, reduced systemic toxicity	unstable in blood, potential for leakage, cleared by the immune system	(136) and (137)
polymeric nanoparticles	paclitaxel, doxorubicin	controlled release, targeted delivery, biodegradable	potential toxicity, immune response, complex design	(138) and (139)
metallic nanoparticles	silver (antibacterial), gold (therapy and imaging)	high drug loading, targeting capability, suitable for thermal therapy	cytotoxicity, organ accumulation, challenges in drug release control	(140−142)
dendrimers	5-fluorouracil, methotrexate	high drug loading capacity, precise molecular architecture, multivalent surface groups	complex synthesis, potential cytotoxicity, costly production	(143−145)
solid lipid nanoparticles	cannabidiol, paclitaxel	good biocompatibility, protects drugs from degradation, controlled release	particle aggregation, production variability, sometimes limited drug loading capacity	(146−148)

Safety is also important, as inadequate delivery systems can cause unwanted side effects or toxicity.^149,150^

Applying bioactive material manufacturing advances to drug delivery can help overcome these challenges by offering more effective and safer delivery systems. For example, nanoparticles, hydrogels, or bioactive polymers can improve drug stability, allowing controlled and targeted release.^151−153^

Customizing delivery systems based on individual patient characteristics can increase treatment efficacy and reduce the risks of side effects.^154^ Ultimately, research in this field has the potential to transform clinical practice by offering more effective and personalized treatments for a variety of medical conditions.^155^

3D printing applied to drug delivery presents significant opportunities and several underexplored areas.^156,157^ Among them is the development of devices for multiphase and personalized drug release, which is essential for complex treatments. The use of natural biomaterials, such as chitosan and collagen, is also promising but remains underutilized despite their bioactive and biocompatible properties.^158,159^ Emerging technologies like 4D printing, which employs programmable materials that respond to stimuli such as pH or temperature, offer new possibilities for intelligent delivery systems but are still in their early stages.^160,161^ Another promising area is the integration of sensors for real-time monitoring and precise drug release control, which requires further exploration.^162,163^

### 3.3 Perspectives, Futures and Gaps

The future of manufacturing bioactive materials for drug delivery not only holds promising advances but also presents several challenges that must be addressed to exploit their potential fully. As the demand for more targeted and efficient drug delivery systems increases, the integration of nanotechnology and biocompatible materials is pivotal. Future perspectives include developing more innovative delivery systems capable of responding to physiological conditions, thereby enhancing the precision of drug release.^164,165^ Additionally, synthesizing bioactive materials that are environmentally sustainable and scalable for industrial production remains a critical gap.^166^ Addressing these challenges involves technological innovation and substantial interdisciplinary collaboration among material scientists, pharmacologists, and regulatory bodies to ensure that these new materials are safe, effective, and economically viable for widespread clinical use. As research progresses, filling these gaps will be essential for advancing personalized medicine and improving patient outcomes globally.

## 4 Conclusions

The development of bioactive materials for drug delivery represents a key frontier in modern medicine, merging innovations in materials engineering with biotechnological advances to create more efficient and precise drug delivery systems. The bibliometric review indicated a significant increase in the volume of publications on this topic, with the journal “Materials” emerging as the most influential, featuring 9 publications and 466 citations.

Bibliometric data also identified China, the U.S., and the U.K. as global leaders in publication volume, citations, and institutional collaboration, with several prominent authors making substantial contributions. The range of publications revealed a rich diversity of applications and a strongly interdisciplinary approach spanning multiple fields of interest.

Keyword network analysis pinpointed key thematic clusters, emphasizing the importance of topics such as regenerative medicine, 3D printing, scaffolds, and biodegradability. Specific areas such as bone regeneration and tissue engineering also stood out.

Moreover, topics such as additive manufacturing, bioactive materials, and 3D printing were explored in depth alongside the primary nanoparticles and drugs used in drug delivery, with a clear assessment of their advantages and disadvantages.

The future of bioactive material fabrication for drug delivery promises greater integration of emerging technologies, such as nanotechnology and 3D printing, driving the development of advanced therapeutic solutions that could revolutionize patient care and optimize clinical outcomes.
